# The Intact Noninducible Latent HIV-1 Reservoir Is Established in an *In Vitro* Primary T_CM_ Cell Model of Latency

**DOI:** 10.1128/JVI.01297-20

**Published:** 2021-03-10

**Authors:** Indra Sarabia, Szu-Han Huang, Adam R. Ward, R. Brad Jones, Alberto Bosque

**Affiliations:** a Department of Microbiology, Immunology and Tropical Medicine, George Washington University, Washington, DC, USA; b Infectious Disease Division, Weill Cornell Medical College, New York, New York, USA; Emory University

**Keywords:** HIV-1 latency, HIV-1 reservoir, HIV-1 subtype, IPDA, latency-reversing agent, shock and kill

## Abstract

The establishment of HIV-1 latency has hindered an HIV-1 cure. “Shock and kill” strategies to target this reservoir aim to induce the latent provirus with latency-reversing agents (LRAs). However, recent studies have shown that the majority of the intact HIV-1 viral reservoir found in antiretroviral therapy (ART)-suppressed HIV-infected individuals is not inducible. We sought to understand whether this noninducible reservoir is established, and thus able to be studied, in an *in vitro* primary T_CM_ cell model of latency. Furthermore, we wanted to expand this model system to include R5-tropic and non-B-subtype viruses. To that end, we generated our T_CM_ cell model of latency with an R5 subtype B virus, AD8, and an R5 subtype C virus, MJ4. Our results demonstrate that both intact and defective proviruses are generated in this model. Less than 50% of intact proviruses are inducible regardless of viral strain in the context of maximal stimulation through the T cell receptor (TCR) or with different clinically relevant LRAs, including the histone deacetylase (HDAC) inhibitors suberoylanilide hydroxamic acid (SAHA) and MS-275, the protein kinase C (PKC) agonist ingenol 3,20-dibenzoate, or the second mitochondria-derived activator of caspase (SMAC) mimetic AZD-5582. Our findings suggest that current LRA strategies are insufficient to effectively reactivate intact latent HIV-1 proviruses in primary CD4 T_CM_ cells and that the mechanisms involved in the generation of the noninducible HIV-1 reservoir can be studied using this primary *in vitro* model.

**IMPORTANCE** HIV-1 establishes a latent reservoir that persists under antiretroviral therapy. Antiretroviral therapy is able to stop the spread of the virus and the progression of the disease but does not target this latent reservoir. If antiretroviral therapy is stopped, the virus is able to resume replication and the disease progresses. Recently, it has been demonstrated that most of the latent reservoir capable of generating replication-competent virus cannot be induced in the laboratory setting. However, the mechanisms that influence the generation of this intact and noninducible latent reservoir are still under investigation. Here, we demonstrate the generation of defective, intact, and intact noninducible latent HIV-1 in a T_CM_ cell model of latency using different HIV-1 strains. Thus, the mechanisms which control inducibility can be studied using this primary cell model of latency, which may accelerate our understanding of the latent reservoir and the development of curative strategies.

## Abstract (precis)

HIV-1 establishes a latent reservoir that persists under antiretroviral therapy. Antiretroviral therapy is able to stop the spread of the virus and the progression of the disease but does not target this latent reservoir.

## INTRODUCTION

Human immunodeficiency virus type 1 (HIV-1) persists during antiretroviral therapy (ART) due to a pool of latently infected cells. ART greatly improves outcomes for people living with HIV-1 (PLWH), but it does not target latently infected cells, so it cannot cure HIV-1 (1–3). It is known that these cells are largely comprised of resting memory CD4 T cells, though other CD4 subsets and other cell types can also contribute to the latent reservoir (4–9). One current strategy toward an HIV-1 cure is “shock and kill,” in which the latent virus is induced using small molecules and subsequently eliminated due to immune clearance or viral cytopathicity (for reviews, see references [Bibr B10][Bibr B11][Bibr B12]). In order to develop successful interventions, the basic mechanisms underlying latency establishment and reversal must be known. Mechanistic studies are difficult to carry out in samples from PLWH due to the availability of sample and the scarcity of these cells *in vivo*; thus, cell lines and primary cell models serve an important purpose in understanding the biology of HIV-1 latency. Our group has extensively characterized a primary T_CM_ cell model of latency that utilizes the replication-competent molecular clone NL4-3, a subtype B CXCR4 (X4)-tropic virus. This model has been extensively used to discover and evaluate latency-reversing agents (LRAs); to perform mechanistic studies examining pathways involved in the establishment and maintenance of latency; and it has several similitudes with latent cells isolated from PLWH, including a similar integration pattern, the presence of clonally expanded integration sites, and similar blocks in HIV-1 splicing (13–29).

Worldwide, the majority of HIV-1 infections are subtype C, and subtype B accounts for a little over ∼10% of total HIV-1 infections (30). Recent studies have highlighted differences in reservoir size among individuals with different subtypes (31, 32). Omondi et al. found that subtype-specific Nef function correlated with reservoir size, but it did not fully explain the differences observed (32). Currently, it is unclear if subtype also plays a role in the establishment of latency or its reversal (for a review, see reference 33). Further, Pierson and colleagues showed that the majority of viruses in the latent reservoir utilize CCR5 (R5) for entry, though some CXCR4 usage was also observed (34). Thus, we wanted to test whether this latency model could be generated using R5-tropic viruses (including a subtype C virus), which may be more biologically relevant to the generation of the latent reservoir and would allow for inclusion of more diverse viruses in HIV-1 cure research using this primary cell model.

Here, we describe such efforts to expand this latency model, thus enhancing its utility for the development of cure strategies and understanding the mechanisms underlying HIV-1 latency. To that end, we characterized the proportions of intact and defective latent proviruses generated in this model with three replication-competent HIV-1 molecular clones and evaluated clinically relevant LRAs for the ability to reactivate latent and intact HIV-1.

## RESULTS

### R5 and subtype C viruses generate latency in the T_CM_ cell model of latency.

**FIG 1:**
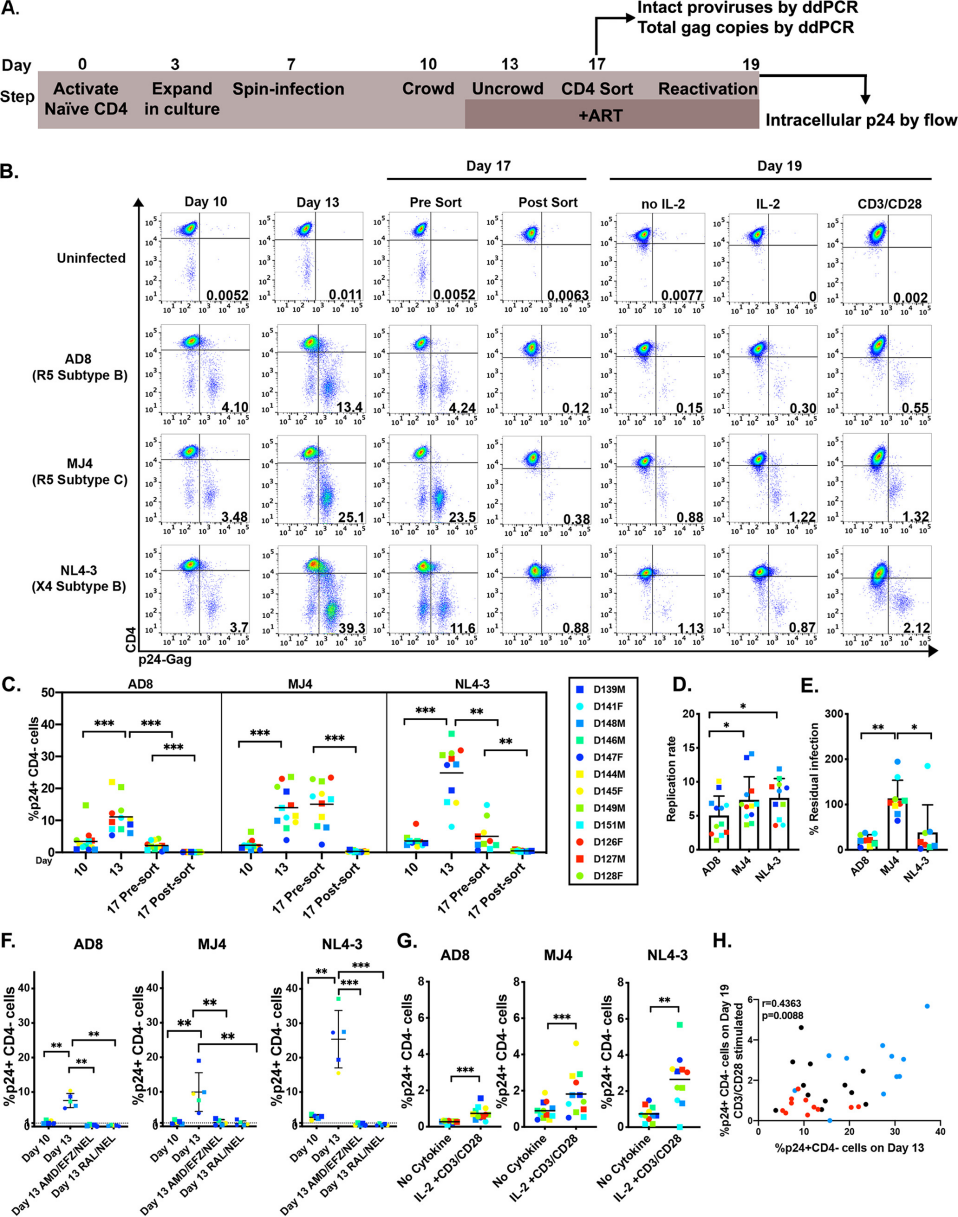
The T_CM_ cell model can be adapted to R5 and subtype C viruses. (A) Outline of the T_CM_ cell model of latency. (B) Percentage of infected cells measured by p24 expression and CD4 downregulation by flow cytometry on days 10, 13, 17, and 19; results for a representative donor are shown. (C) Summary of the primary cell model generated with 11 or 12 donors per virus. (D) Analysis of the rate of viral replication from day 10 to day 13. (E) Analysis of residual infection at day 17 after addition of ARV on day 13. (F) Percentage of cells infected on days 10 and 13, with or without addition of ARV combinations AMD-3100/efavirenz (EFZ)/nelfinavir (NEL) or raltegravir (RAL)/nelfinavir. (G) Measurement of latency reversal with no cytokine or IL-2/CD3/CD28 stimulation for 48 h; reactivated cells were measured by p24 expression and CD4 downregulation. (H) Correlation between percentage of infected cells on day 13 and reactivation on day 19 with CD3/CD28 beads, calculated using nonparametric Spearman correlation. AD8 is labeled red, MJ4 is black, and NL4-3 is blue. Circles indicates female blood donors, and squares indicate male blood donors. Wilcoxon matched-pairs signed rank test was used to calculate *P* values (*n* = 11 or 12 donors per virus). *, *P* < 0.05; **, *P* < 0.01; ***, *P* < 0.001.

To expand this latency model to include an R5 or a non-B-subtype virus, we chose the R5 subtype B virus AD8 (35) and the R5 subtype C virus MJ4 (36) to generate latently infected cells using the cultured T_CM_ cell model, as outlined in Fig. 1A (18, 19). Expanded naïve CD4 T cells were spin-infected with either AD8, MJ4, or NL4-3 at a low multiplicity of infection. We measured productive infection *in vitro* on day 10 using flow cytometry by staining for intracellular p24-Gag expression and surface CD4 downregulation (Fig. 1B, day 10, and Fig. 1C). We then “crowded” the cells to facilitate cell-to-cell spread of infection (Fig. 1B, day 13, and Fig. 1C). We consistently observed an increase in infection from day 10 to day 13, across multiple donors, showing that the R5 viruses AD8 and MJ4 were able to infect and replicate *in vitro* in this model (Fig. 1C). There were significant differences in replication rates (change in infection from day 10 to day 13) between AD8 and NL4-3 and between AD8 and MJ4, where AD8 had the lowest replication rate (Fig. 1D). After uncrowding, cells were cultured in the presence of AMD-3100, efavirenz, and nelfinavir to stop the spread of infection for an additional 4 days. We previously published this model using raltegravir and nelfinavir but eliminated the integrase inhibitor raltegravir from our culturing conditions in this study to reduce 2-long terminal repeat (2-LTR) circle accumulation (24). After 4 days in culture with antiretroviral drugs (ARVs), productively infected cells decreased for AD8 and NL4-3 (Fig. 1B, day 17 presort, and Fig. 1C and E). We did observe a greater percentage of MJ4-infected cells remaining in culture after ARV introduction. The new combination of ARVs is equally as effective at suppressing viral replication as the original combination of raltegravir and nelfinavir for all strains, indicating that the remaining HIV-1-infected cells were not due to ongoing viral replication (Fig. 1F).

Currently, there is no biomarker for viral latency, so we enriched for latently infected cells by eliminating the productively infected cells from culture. To do this, cells were magnetically sorted based on CD4 expression on day 17, which eliminated productively infected cells measured as p24 positive and CD4 negative (Fig. 1B, day 17 postsort). CD4 expression is downregulated on the cell surface due to the expression of the accessory Nef and Vpu genes (37, 38). This procedure leaves only uninfected and latently infected cells (19). We eliminated productively infected cells to assess *de novo* reactivation from latency without the confounding variable of ongoing viral replication. To reactivate latent proviruses, cells were stimulated with either ARVs and interleukin 2 (IL-2)/CD3/CD28, which mimics T cell activation, or an ARV-containing medium control for 48 h. All viruses established latency and were reactivated; AD8 exhibited the lowest percentage of reactivated cells when stimulated with IL-2/CD3/CD28, followed by MJ4 and then NL4-3 (Fig. 1G). In this model of latency, we had previously observed that the degree of infection on day 13 was correlated with the degree of reactivation seen on day 19 with CD3/CD28 beads (18). Indeed, we observed a correlation between infection at day 13 and reactivation on day 19 with all viruses used (Fig. 1H). Our measure of viral reactivation, p24-Gag expression and CD4 downregulation by flow cytometry, takes into account transcription, splicing, and translation of viral proteins but does not reveal the total size of the pool of latently infected cells able to be reactivated, thus raising the question of whether all possible proviruses are successfully reactivated in this *in vitro* model. To address this question, we used measurement of proviral DNA to estimate the pool of potentially inducible latently infected cells.

### Low inducibility of latent HIV-1 despite maximal stimulation.

**FIG 2:**
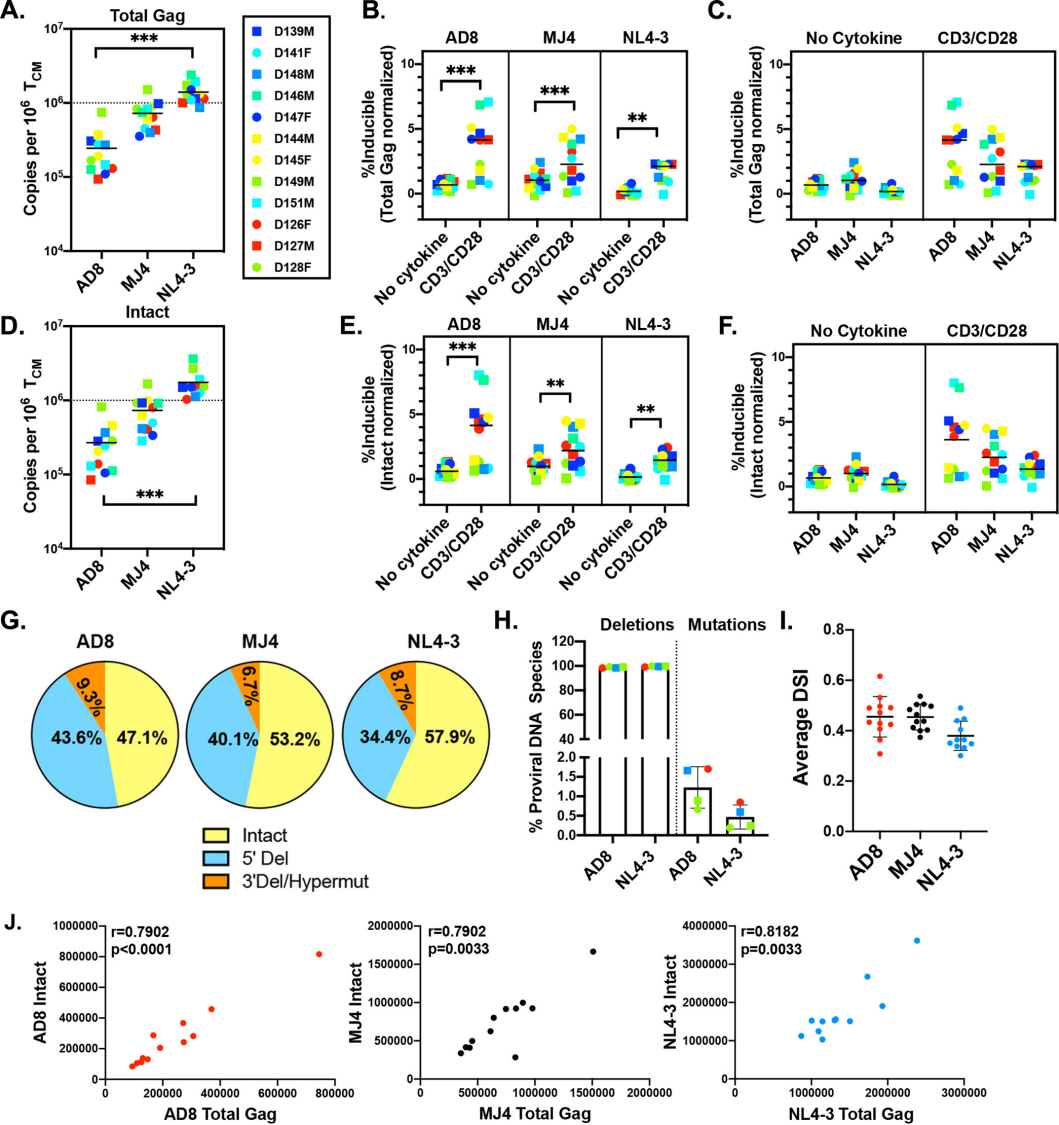
Latently infected cells exhibit low inducibility despite maximal stimulation. (A) Total HIV-1 *gag* copies per million cultured T_CM_ cells. The percentage of inducible proviruses was determined by dividing the CD3/CD28-stimulated reactivated cells per million cultured T_CM_ cells by the total *gag* HIV-1 copies per million cultured T_CM_ cells, separated by virus (B) or stimuli (C). (D) Total copies of intact proviruses were determined using the IPDA by digital droplet PCR. (E and F) The percentage of intact inducible proviruses was determined by dividing the CD3/CD28-stimulated reactivated cells per million cultured T_CM_ cells by the intact HIV-1 copies per million cultured T_CM_, separated by virus (E) or stimuli (F). (G) Proportions of 5′-deleted, 3′-deleted or -hypermutated, and intact proviruses determined by IPDA; mean percentages of each species for each virus are reported. (H) Subset of donors assessed for deletion or hypermutation of the 3′ region in modified IPDA. (I) Average DNA shearing index for DNA samples; standard deviation is shown. (J) Correlation of total HIV-1 *gag* copies per million cultured T_CM_ cells and total intact HIV-1 copies per million cultured T_CM_ cells, calculated using nonparametric Spearman correlation. Dunn’s multiple-comparison test was used to calculate *P* values for panels A, C, D and G, and Wilcoxon matched-pairs signed rank test was used for panels B and F (*n* = 11 or 12). **, *P* < 0.01; ***, *P* < 0.001.

After sorting based on CD4 expression at day 17 and prior to viral reactivation, DNA was isolated and total HIV-1 *gag* copies were quantified using digital droplet PCR (ddPCR). Total *gag* copies were normalized to T_CM_ cells by measuring copies of RPP30 by ddPCR. AD8 had the lowest copies of HIV-1 *gag*, followed by MJ4 and then NL4-3 (Fig. 2A). After normalizing the viral reactivation shown in Fig. 1G to total HIV-1 *gag* copies, we still observed a statistically significant reactivation from latency with IL-2/CD3/CD28 stimulation for all viruses (Fig. 2B). Interestingly, we observed that IL-2/CD3/CD28 stimulation induced less than 7% of total HIV-1 *gag* copies regardless of viral strain. There were no significant differences in reactivation between any of the viruses (Fig. 2C). It has been previously shown that the majority of proviruses are defective in CD4 T cells isolated from PLWH on long-term ART (39). It is possible that we observed low inducibility as a result of normalizing to total HIV-1 *gag* copies, many of which could be defective, thus overestimating the pool of potentially inducible cells (40). To address this concern, we characterized the composition of intact versus defective proviruses in this *in vitro* model, in order to normalize reactivation to copies of intact proviruses. We used a modified version of the intact proviral DNA assay (IPDA) to determine whether proviruses were intact or deleted/mutated (41). We observed the same patterns as with total HIV-1 *gag* copies, where AD8 had the fewest intact proviral DNA copies, followed by MJ4 and then NL4-3 (Fig. 2D). We observed the same patterns of inducibility when the results were normalized to total intact proviral DNA copies instead of total HIV-1 *gag* copies (Fig. 2E) and did not observe a significant difference between viruses (Fig. 2F). Despite the short culture time, 47 to 58% of proviruses were intact and 43 to 53% of proviruses were defective in the 5′ or 3′ region of the HIV-1 genome (Fig. 2G), the majority of them being 5′ deletions. In a subset of donors, we observed that most of 3′ defective viruses were due to deletions and not hypermutations (Fig. 2H). The DNA shearing index (DSI) used to account for shearing in the IPDA is shown in Fig. 2I and is similar to what has been previously published (41). In this model, the copies of intact proviruses and total HIV-1 *gag* copies are correlated for all viruses used (Fig. 2J).

### LRAs are ineffective at inducing the majority of intact latent proviruses.

**FIG 3:**
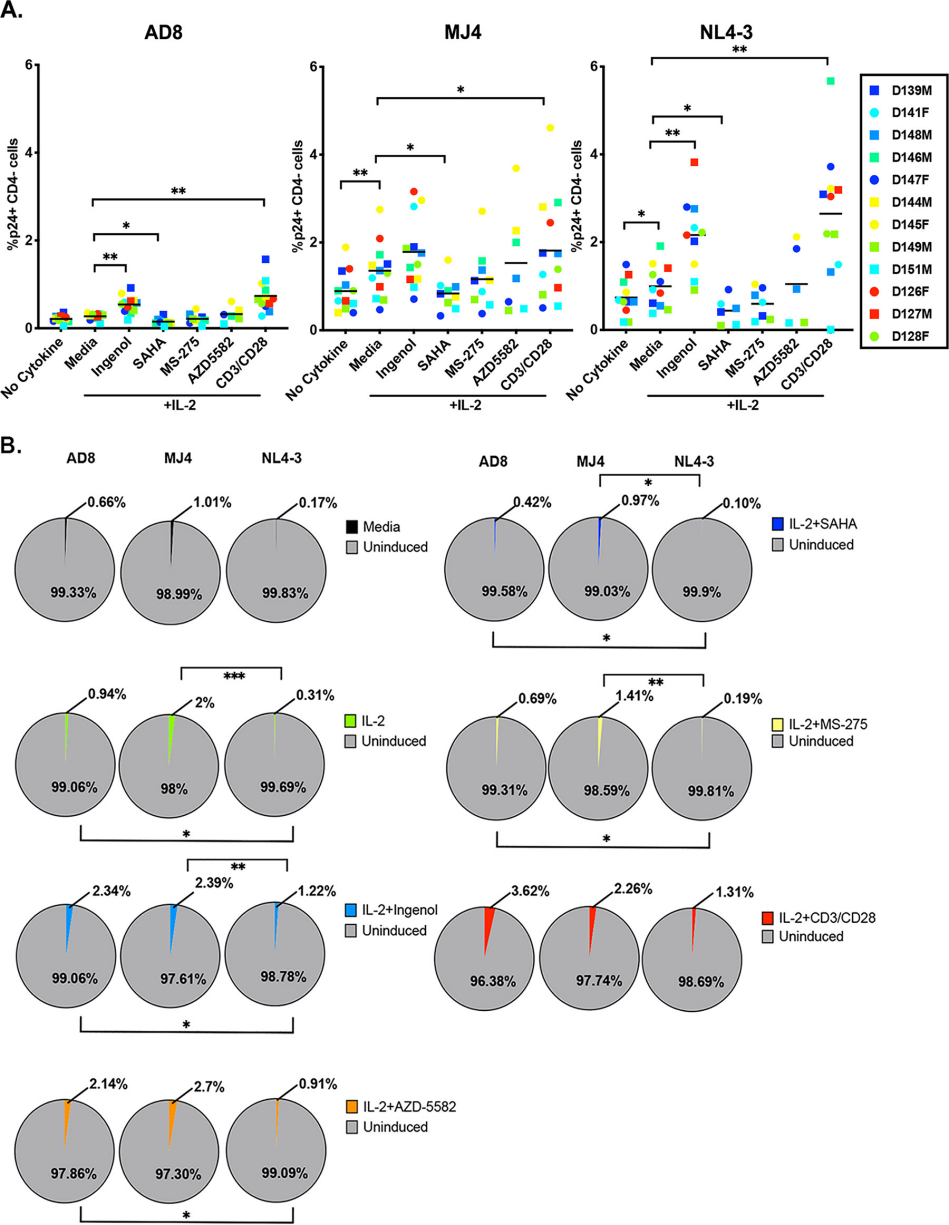
Clinically relevant LRAs are largely ineffective at reactivating intact latent proviruses. (A) A panel of LRAs were tested in the ability to reactivate latent HIV in latently infected cells generated with AD8, NL4-3, or MJ4. Reactivation was measured by p24 expression and CD4 downregulation by flow cytometry. Wilcoxon matched-pairs signed rank test was used to calculate *P* values and adjusted using Holm’s step-down method. (B) Percentage of intact induced proviruses was determined by dividing the LRA-specific reactivated cells per million CD4 cells by the intact HIV-1 copies per million CD4 cells. Dunn’s multiple-comparison test was used to calculate *P* values (*n* = 5 to 12 depending on LRA). *, *P* < 0.05; **, *P* < 0.01; ***, *P* < 0.001.

We next addressed LRA activity in this latent cell model generated with the 3 distinct HIV-1 viruses. We selected a panel of LRAs from three distinct classes: the protein kinase C (PKC) agonist ingenol 3,20-dibenzoate (ingenol) (42, 43), histone deacetylase (HDAC) inhibitors (HDACi) SAHA and MS-275 (44–48), and the SMAC mimetic AZD-5582 (49, 50). At day 17, sorted latently infected cells were stimulated with LRAs for 48 h with the exception of AZD-5582, which was added for 1 h and then washed out (49, 50). We chose 48-h stimulation to assess the efficacy of reactivation and compare to previously published works that have characterized the LRA activity of these compounds at this time point (42, 43, 48, 49). IL-2 alone was sufficient to reactivate MJ4 and NL4-3 latently infected cells (Fig. 3A). Ingenol and AZD-5582 activate the canonical and noncanonical nuclear factor kappa light chain enhancer of activated B cells (NF-κB) signaling pathway, respectively. Ingenol significantly reactivated AD8 and NL4-3 latently infected cells but was not significantly different than the IL-2 control for MJ4 (Fig. 3A). AZD-5582 reactivated latent HIV-1, though it did not reactivate more than CD3/CD28 in 3/6 (AD8), 3/7 (MJ4), and 3/6 (NL4-3) latently infected donors (Fig. 3A). HDACi reduced the ability of IL-2 to reactivate latent HIV-1 in this model of latency. We next wanted to assess how effective these LRAs were at inducing the intact latent reservoir, so we normalized the data to intact copies of HIV-1 (Fig. 3B). The mean percentage of inducible intact proviruses by each individual LRA was less than 3%, while 97% of the intact latent reservoir was unperturbed in this model of latency; no single LRA reactivated more than CD3/CD28 stimulation. We observed minor differences between viruses with respect to sensitivity to the three distinct classes of LRAs.

### The intact, integrated, inducible reservoir in the T_CM_ cell model of latency.

**FIG 4:**
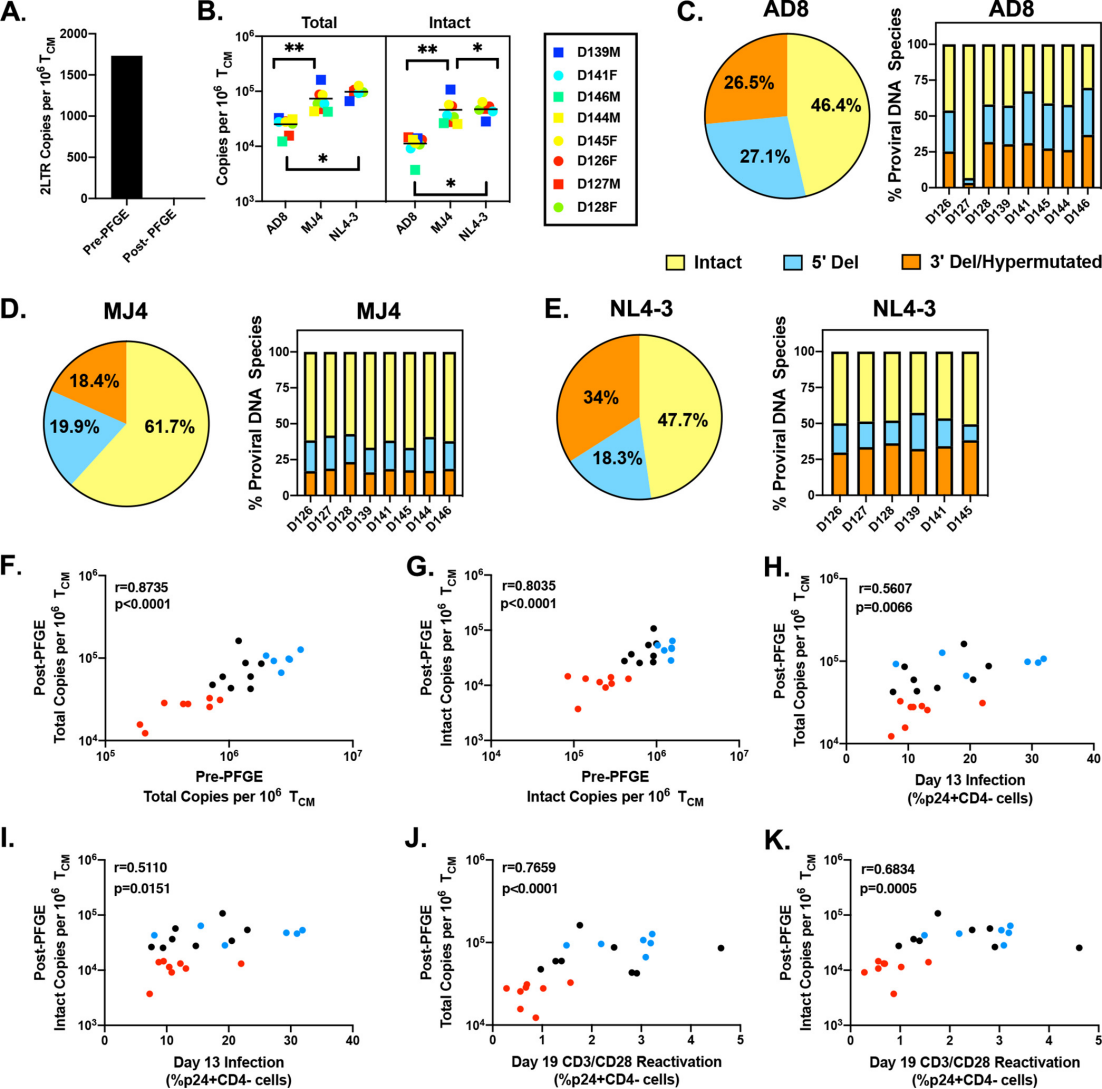
Isolation of HMW DNA eliminates unintegrated proviruses and reveals greater inducibility in the T_CM_ cell model of latency. (A) Measurement of 2-LTR circles in a single donor pre- and post-PFGE. (B) Total HIV-1 DNA copies (sum of 5′-deleted, 3′-deleted/hypermutated, and intact proviruses) and intact HIV-1 DNA copies measured in samples post-PFGE. Proportions of intact, 5′-deleted, and 3′-deleted/hypermutated proviruses are shown as averages of all donors in the pie chart and individual donors for each virus: AD8 (C), MJ4 (D), and NL4-3 (E). (F and G) Correlation between pre- and post-PFGE total HIV-1 DNA copies (F) or intact HIV-1 DNA copies (G). (H and I) Correlation between day 13 infection and post-PFGE total HIV-1 DNA copies (H) or intact (I). (J and K) Correlation between reactivation on day 19 with CD3/CD28 beads and post-PFGE total HIV-1 DNA (J) or intact HIV-1 DNA (K). Correlations were calculated using nonparametric Spearman correlation. AD8 is labeled red, MJ4 is labeled black, and NL4-3 is labeled blue for panels F to K. *, *P* < 0.05; **, *P* < 0.01.

**FIG 5:**
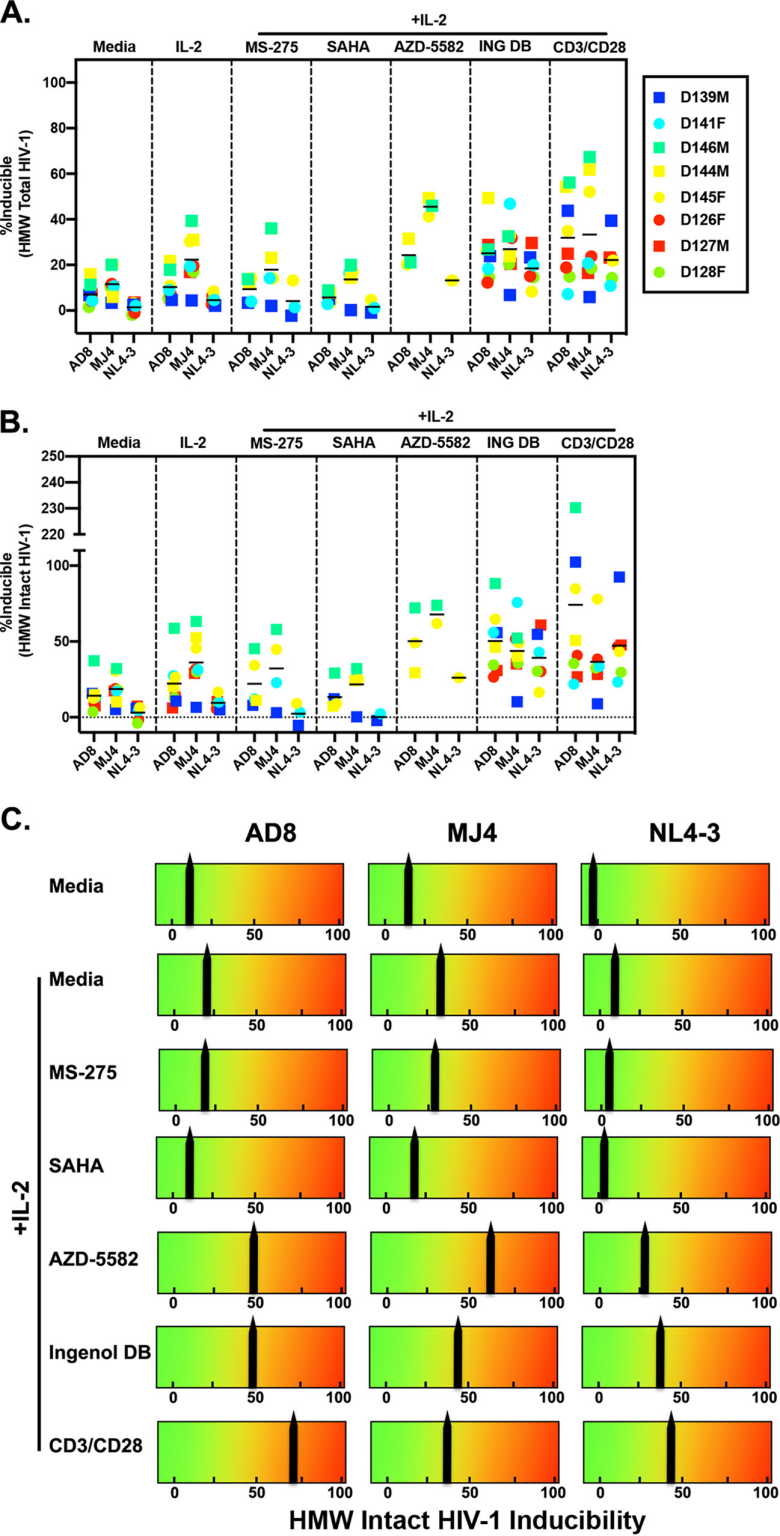
LRAs do not reactivate majority of integrated intact proviruses in this *in vitro* system. (A and B) Reactivation for each LRA condition measured by percentage of p24 expression and CD4 downregulation by flow cytometry, normalized to post-PFGE total (intact, 5′-deleted, and 3′-deleted/hypermutated) HIV-1 DNA copies (A) or post-PFGE intact HIV-1 DNA copies (B). (C) Average reactivation per LRA and per virus.

Although our culturing conditions did not include raltegravir, which could cause an accumulation of 2-LTR circles, we determined whether the intact copies measured by ddPCR were also integrated. Linear and unintegrated HIV-1 DNA could potentially be a bias in downstream DNA assays. We used pulsed-field gel electrophoresis (PFGE) to remove unintegrated HIV-1 DNA from a subset of our samples. PFGE has been shown to effectively eliminate unintegrated HIV-1 DNA and is correlated with Alu PCR (51, 52), the gold standard for measuring integrated HIV-1 DNA. We confirmed that PFGE removed 2-LTR circles (Fig. 4A) as previously reported (51, 52). We then repeated the IPDA and measured the number of total (sum of intact and defective proviruses) and intact copies after PFGE (Fig. 4B). The percentages of total copies that remained after PFGE were as follows: AD8 had an average of 6% ± 2.1%, MJ4 had an average of 6.1% ± 3.3%, and NL4-3 had 3.6% ± 1%. The percentages of intact copies that remained after PFGE were as follows: AD8 had, on average, 6.4 ± 4.9%, MJ4 had 6.1% ± 2.8%, and NL4-3 had 3.1% ± 1.1%. With all three viruses, overall, 5.5% ± 3.5% of the intact copies remained after PFGE, and 5.4% ± 2.6% of total copies remained after PFGE. After PFGE, AD8 had the smallest percentage of intact copies and similar proportions of 5′-deleted and 3′-deleted/hypermutated copies (Fig. 4C). MJ4 had the greatest proportion of intact proviruses, with similar distributions of defective proviruses (Fig. 4D). Interestingly, NL4-3 had similar levels of intact proviruses as AD8 but had more 3′-deleted/hypermutated copies than 5′-deleted copies (Fig. 4E). We found a correlation between total copies (Fig. 4F) as well as intact copies (Fig. 4G) of HIV-1 pre- and post-PFGE. Similar to our previous data with total HIV-1 Gag copies, we also observed a correlation between infection at day 13 and total HIV copies (Fig. 4H) and intact copies (Fig. 4I) post-PFGE. In our post-PFGE samples, we also observed a correlation between reactivation seen with CD3/CD28 stimulation on day 19 and total HIV-1 copies (Fig. 4J) and intact HIV-1 DNA copies (Fig. 4K). When reactivation was normalized to total integrated proviruses, we observed that an average of 30% of the latent reservoir generated in this latency model was induced with CD3/CD28 stimulation (Fig. 5A) and an average of 50% reactivation when normalized to integrated intact copies (Fig. 5B). We observed similar patterns of reactivation with LRAs as before, but the magnitude of induction was greater. No single LRA exceeded the reactivation induced by CD3/CD28, though ingenol and AZD-5582 (in a subset of donors) were the most potent of the clinically relevant LRAs tested (Fig. 5C).

## DISCUSSION

*In vitro* latency models are important tools in the development of HIV-1 cure strategies. Prior to this study, the T_CM_ cell latency model had been generated only with CXCR4-tropic subtype B viruses. The majority of viruses in the latent reservoir utilize CCR5 for entry (34), providing a strong rationale to generate this model using R5 viruses. Our data show that this model can generate a heterogenous latent reservoir *in vitro*, which harbors intact, integrated, inducible, and noninducible latent proviruses with an R5 or subtype C virus. AD8 is a virus with an R5 primary isolate envelope (from HIV-1 ADA derivative AD8.1), with the backbone of NL4-3 (35). During the generation of these latently infected cells, we observed differences in infection between AD8 and NL4-3, likely due to differences in infection efficiency between virus envelopes (53, 54) and/or less CCR5 expression than CXCR4 expression in this model (14). As both R5 viruses infected, replicated, and established latency in this model, it is a proof of concept that using more diverse R5-tropic viruses is possible within this system. Further, we recently showed that this model allows the generation of latently infected cells using primary viruses isolated from ART-suppressed PLWH using the quantitative viral outgrowth assay (QVOA) (26).

Interestingly, in this model almost half of proviruses are defective despite a short replication time (7 days). Defective proviruses are most abundant in CD4 T cells isolated from PLWH on long-term ART (39, 52, 55, 56). Specifically, in the T_CM_ subset, 3′ deletions/hypermutations are most abundant, followed by 5′ deletions and, lastly, intact copies (41). This is in contrast to our *in vitro* model, which had, on average, ∼50% intact proviruses, followed by similar proportions of 3′-deleted/hypermutated and 5′-deleted proviruses across all viruses used. However, our results are in line with previous observations on intact proviruses generated *in vitro*. Pinzone and colleagues characterized intact and defective provirus kinetics in their *in vitro* model and found that resting cells accumulated more defective proviral forms, but in their single round of infection, the majority of copies were intact (52). In our latency model, we infect cells 7 days after activation, as they are transitioning into a more resting phenotype, which may explain why we observe defective forms and why a much larger portion of latent proviruses are intact than in CD4 T cells isolated from PLWH on long-term ART. Additionally, the composition of intact and defective proviruses may be affected by the short culturing time frame, the lack of selection due to immunological responses to defective viruses (57), or the shorter period of infection, thus explaining the minor proportion of APOBEC3G-induced mutations or deletions (58–60). With regard to proportions of intact proviruses between the three viruses tested, we observed a trend in which MJ4 generated the most intact proviruses (post-PFGE). However, further work with other subtype C clones or primary viral isolates is needed to determine whether this is intrinsic to that particular HIV-1 molecular clone or whether it is subtype specific. We observed that in some donors we measured over one copy of HIV-1 per cell when measuring total HIV Gag, suggesting multiple proviruses per cell or unintegrated forms present in the sample. Indeed, after PFGE we found that 91% to 97% of those copies detected were unintegrated, but the majority of unintegrated forms were not explained by 2-LTR circles alone, as they represent ∼0.2% of copies in a representative sample pre-PFGE (Fig. 4A). This proportion of integrated copies is consistent with a previous study in which approximately only 10% of HIV-1 DNA copies were detected using Alu PCR (61). Future work in this *in vitro* model will need to eliminate unintegrated HIV-1 copies before drawing conclusions on provirus inducibility, reduction of the latent reservoir, or infection frequency using any DNA-based measurement.

Due to donor-to-donor variation, it is not possible to get the same degree of latent infection each iteration of the model. Therefore, HIV-1 DNA measurements are required to estimate the pool of latently infected cells. Total HIV-1 Gag or total intact copies could overestimate the size of the latent reservoir unless nonintegrated forms are eliminated. Once reactivation was normalized to post-PFGE intact copies, we observed that an average of 50% of intact integrated proviruses were induced with CD3/CD28. This is in contrast to the low inducibility observed in CD4 T cells isolated from PLWH on long-term ART (41), likely due to the stark differences in proportions of intact proviruses between the two types of samples, selection of noninducible proviruses over time in PLWH, or cell-intrinsic differences. Interestingly, in a subset of donors the percent inducibility is over 100% with CD3/CD28 stimulation. This is a potential caveat of the study, since it could mean that there are somehow more reactivated cells than infected cells. One possible explanation is that some defective proviruses may still be able to express Nef and Gag proteins (56) and are captured in our p24-CD4 flow assay. Indeed, when we normalized to total (the sum of intact, 5′-defective, and 3′-defective/hypermutated copies) post-PFGE HIV-1 copies, we did not observe any donors above 100% inducibility. Since it is not possible at this time to simultaneously distinguish which cells reactivated because of reactivation of an intact latent provirus versus a defective latent provirus, an additional measurement of viral release or viral outgrowth after stimulation may be helpful in determining inducibility more accurately.

With that caveat in mind, the LRAs assessed in this work were not more effective than CD3/CD28 stimulation, which mainly triggers nuclear factor of activated T cells (NFAT) in this model (14). Of the LRAs tested, ingenol 3,20-dibenzoate and AZD-5582 came closest in activity to CD3/CD28 (Fig. 5C). Ingenol 3,20-dibenzoate triggers the canonical NF-κB pathway (42), while AZD-5582 triggers the noncanonical NF-κB pathway (49). We observed better reactivation of MJ4 latently infected cells to AZD-5582 than other classes of LRAs tested when comparing to AD8 or NL4-3 in magnitude of reactivation. This could be due to subtype-specific factors. Subtype C LTR can harbor 3 or 4 NF-κB binding sites, instead of 2 as found in subtype B; this could explain a higher response to certain stimuli that activate NF-κB (33). However, further work needs to be done with other subtype C clones or primary viral isolates to determine whether this is intrinsic to that particular HIV-1 molecular clone or whether it is subtype specific. Previous work in cell lines has shown that additional NF-κB sites lead to enhanced transcriptional activity (62, 63), but this has not been evaluated in primary cell models with replication-competent viruses. We did not observe LRA activity with SAHA. This may be explained because we measured viral protein expression upon reactivation, and previous works describing these LRAs have measured mostly viral RNA production (46, 47, 64). MS-275 did not have activity in reactivating latent HIV-1 in our latency model either. Taken together, these data show that current LRAs do not reactivate the majority of the latent reservoir generated in this system and would likely be ineffective for HIV-1 cure strategies as single agents.

Our study is not without caveats: this *in vitro* model is a T_CM_ cell-based model of latency and thus cannot recapitulate how other T cell subsets or cell types of the latent reservoir may behave. Although it has been previously shown that distinct CD4 T cell subsets have differing degrees of sensitivity to latency reversal (65–67), recent work by Kwon et al. has shown that intact proviruses are distributed evenly among the CD4 T cell subsets and are similarly poorly inducible (68). This *in vitro* system was designed to mimic latently infected T_CM_ cells from blood, which are a suitable proxy for reservoir measurement in secondary lymphoid organs such as lymph nodes (69). It is important to note that this model uses only CD4 T cells; consequently, this model cannot recapitulate immune selective pressures on the virus that occur *in vivo* (57).

In spite of these caveats, this model has several similitudes with the reservoir in PLWH. First, as we show in this work, this model generates defective and intact proviruses, some of which are not induced with maximal stimulation *in vitro* similar to that found CD4 T cells isolated from PLWH on long-term ART. Second, this model recapitulates similar integration patterns, including the generation of expanded sites (23). Third, this model recapitulates the blocks to HIV-1 transcription initiation, multiple splicing, and potentially elongation that are observed in CD4 T cells isolated from PLWH on long-term ART (26). These similarities are likely due to the use of replication-competent viruses and primary cells, as their metabolism and abundance of host factors better mirror those of primary CD4 T cells than of cell lines.

The main conclusions from this study are that the T_CM_ cell latency model can indeed be expanded to use R5 and non-subtype-B virus strains, increasing the utility of this model for HIV-1 cure strategies, as the majority of the worldwide HIV-1 infections are non-B (30). This model also generates a heterogenous reservoir with intact and defective proviruses despite a short culturing time as well as recapitulating the generation of intact noninduced proviruses. Therefore, this model could be used to further understand the mechanisms involved in HIV-1 persistence in CD4 T cells as well as for the preclinical evaluation of HIV-1 cure strategies.

## MATERIALS AND METHODS

### Reagents.

The following reagents were obtained from the NIH AIDS Research and Reference Reagent program, Division of AIDS, NIAID: nelfinavir, AMD-3100, and efavirenz. HIV-1_NL4-3_ was obtained from Malcolm Martin (catalog no. 114) (70). The HIV-1 NL4-3_AD8_ infectious molecular clone [pNL(AD8)] was obtained from Eric O. Freed (catalog no. 11346) (35). The HIV-1_MJ4_ infectious molecular clone (pMJ4) was obtained from Thumbi Ndung’u, Boris Renjifo, and Max Essex (catalog no. 6439) (36). Recombinant IL-2 was obtained from the NCI preclinical repository. SAHA and MS-275 were obtained from Cayman Chemical. AZD-5582 was obtained from Selleck Chem. Ingenol 3,20-dibenzoate was obtained from Enzo Life Sciences. Αnti-CD3/anti-CD28-coated beads (Dynabeads) were obtained from Invitrogen.

### Generation of latently infected T_CM_ cells.

Latently infected cells were generated as previously described (14, 15, 18, 19). Samples from both male and female blood donors were used in the generation of latently infected cells as previously described (13–15, 18, 19), with some modifications. Briefly, naive CD4 T cells were isolated from HIV-1-negative blood donors using magnetic isolation (Sepmate primary human CD4 T cell isolation kit, STEMCELL Technologies). Naive CD4 T cells were activated with anti-CD3/anti-CD28 Dynabeads (1:1 ratio of cells to beads) in the presence of anti-human IL-4, anti-human IL-12, and transforming growth factor β1 (TGF-β1) (1 μg/ml, 2 μg/ml, and 10 ng/ml, respectively, from Peprotech). Cells were plated in 96-well round-bottom plates at a density of 0.5 × 10^6^/ml in RPMI medium supplemented with 10% fetal bovine serum (FBS), penicillin/streptomycin, and l-glutamine (complete RPMI medium) for 3 days. Afterwards, anti-CD3/anti-CD28 beads were removed using a Dynal MPC-L magnetic particle concentrator (Invitrogen). Cells were resuspended and kept at a density of 1 × 10^6^/ml in complete RPMI medium with 30 IU/ml of IL-2. Medium was replaced on days 4 and 5 of culture. To generate latently infected cells, cells were infected on day 7 of culture using NL-AD8 (referred to here as AD8) and MJ4 in addition to NL4-3. One-fifth of the culture was kept uninfected in complete medium with IL-2, and one-fifth of the cells was infected with either AD8, MJ4, or NL4-3 by spinoculation at 2,900 rpm for 2 h at 37°C. The amount of virus used for infection was determined by titrating in day 7 primary CD4 T cells to achieve around 3 to 5% infection at day 10. After spinoculation, infected cells were added to the remaining three-fifths of culture with complete medium and IL-2. At day 10, cells were plated in 96-well round-bottom plates in complete medium with 30 IU/ml IL-2 to facilitate cell-to-cell spread of infection (“crowding” phase). At day 13, cells were transferred to flasks and the following antiretroviral drugs were added to both infected and uninfected cultures to stop further infection: 0.5 μM nelfinavir, 100 nM efavirenz, and 100 nM AMD-3100. At day 17, infected and uninfected cells were sorted using a CD4 positive isolation kit (Dynabeads, 11331D; Invitrogen) to isolate the latently infected cell population. The isolation was carried out as indicated in the manufacturer’s protocol, with two modifications: (i) the amount of CD4 beads was increased 3-fold and (ii) the resuspension volume of buffer II was changed to 200 to 300 μl per 10^7^ cells.

### Isolation of high-molecular-weight DNA using PFGE.

Genomic DNA was extracted using the DNeasy blood and tissue kit (Qiagen) according to the manufacturer’s protocol from a minimum of 1 × 10^6^ day 17 CD4-sorted latently infected cells. A portion of the isolated genomic DNA was set aside for droplet digital PCR (ddPCR) assays described below, while the remaining genomic DNA was subjected to pulsed-field gel electrophoresis. High-molecular-weight (HMW) DNA (which is enriched for integrated HIV-1 DNA) was isolated using the BluePippin platform (Sage Science) as previously described (51, 52), with one modification: the cutoff for DNA collection was lowered to 10 kb to improve DNA yield. Purified high-molecular-weight DNA was then used for ddPCR assays to determine intact, integrated proviruses.

### ddPCR.

Genomic DNA from day 17 latently infected cells was isolated as described above. For each PCR, 50 ng of DNA fragmented using QiaShredder columns (Qiagen) or up to 50 ng of purified HMW DNA was used directly. DNA was added to ddPCR Supermix for probes (Bio-Rad) with a 900 nM final concentration of primers and a 250 nM final concentration of probes. Droplets were generated using the QX100 droplet generator (Bio-Rad). For total HIV-1 *gag* copies, plates were cycled as previously described (71) and read on a QX100 droplet reader (Bio-Rad). Most primers and probes were previously published (71), but those generated in this study are listed in Table 1. The IPDA was performed as previously described with previously published primers (41), with a few modifications. The thermal cycling parameters were as follows: 95°C for 10 min for 1 cycle, then 94°C for 30 s and 53°C for 1 min for 40 cycles, followed by 98°C for 10 min for 1 cycle, with maintenance at 12°C until reading. The DNA shearing index was calculated and applied to the copies of intact proviruses and proviruses with hypermutations or deletions as previously described (41). For subtype C samples, primers and probes were designed to span the same packaging signal and envelope regions as the subtype B IPDA primers (Table 1). RPP30 was used to normalize *gag* or intact proviral DNA copies to cell numbers. To distinguish between 3′ deletions or hypermutations, a modified IPDA was performed by using the envelope primers and probe with a fluorescent hypermutation probe in the same reaction.

**TABLE 1:** Primer and probe sequences for ddPCR assays

Primer/probe name	Sequence	Reference
RPP30 F	GATTTGGACCTGCGAGCG	71
RPP30 R	GCGGCTGTCTCCACAAGT	71
RPP30 probe	VIC-CTGAACTGAAGGCTCT-MGBNFQ	71
HIV-gag NL4-3 F	TCTCGACGCAGGACTCG	This paper
HIV-gag NL43 R	TACCGACGCTCTCGCACC	This paper
HIV-gag MJ4 F	TCTCGACGCAGGACTCG	This paper
HIV-gag MJ4 R	TATTGACGCTCTCGCACC	This paper
RPP30 Shear 1 F	CCATTTGCTGCTCCTTGGG	This paper
RPP30 Shear 1 R	CATGCAAAGGAGGAAGCCG	This paper
RPP30 Shear 1 Probe	/56-FAM/AAGGAGCAA/ZEN/GGTTCTATTGTAG/3IABkFQ/	This paper
RPP30 Shear 2 F	GATTTGGACCTGCGAGCG	This paper
RPP30 Shear 2 R	GCGGCTGTCTCCACAAGT	This paper
RPP30 Shear 2 Probe	VIC-CTGACCTGAAGGCTCT	This paper
IPDA SubtypeB Psi F	CAGGACTCGGCTTGCTGAAG	41
IPDA SubtypeB Psi R	GCACCCATCTCTCTCCTTCTAGC	41
IPDA SubtypeB Psi Probe	/56-FAM/TTTTGGCGT/ZEN/ACTCACCAGT/3IABkFQ/	41
IPDA SubtypeB Env F	AGTGGTGCAGAGAGAAAAAAGAGC	41
IPDA SubtypeB Env R	GTCTGGCCTGTACCGTCAGC	41
IPDA SubtypeB Env Probe	/5HEX/CCTTGGGTT/ZEN/CTTGGGA/3IABkFQ/	41
Env Hypermutation Probe nonfluorescent	/5IABkFQ/CCTTAGGTTCTTAGGAGC/3IABkFQ/	41
Env Hypermutation Probe fluorescent	/56-FAM/CCTTAGGTT/ZEN/CTTAGGAGC/3IABkFQ/	41
IPDA SubtypeC Psi F	GGACTCGGCTTGCTGAAGTG	This paper
IPDA SubtypeC Psi R	CACCCATCTCTCTCCTTCTAGCC	This paper
IPDA SubtypeC Psi Probe	/56-FAM/TGGTGAGTA/ZEN/CGCCAAATTT/3IABkFQ/	This paper
IPDA SubtypeC Env F	AGTGGTGGAGAGAGAAAAAAGAGC	This paper
IPDA SubtypeC Env R	GTCTGGCCTGTACCGTCAGC	This paper
IPDA SubtypeC Env Probe	/5HEX/CCTTGGGTT/ZEN/CTTGGGA/3IABkFQ/	This paper

### Reactivation assays.

For latency reversal assays, 1 × 10^5^ to 3 × 10^5^ latently infected cells (day 17, CD4 sorted) were plated in ARV-containing media (AMD-3100, nelfinavir, and efavirenz) with 30 IU of IL-2 and treated with the desired LRAs for 48 h with the exception of AZD5582: 100 nM ingenol 3,20-dibenzoate, 330 nM SAHA, or 10 μM MS-275. For AZD5582 stimulation, cells were incubated with 100 nM AZD5582 for 1 h and then washed to remove the compound and avoid toxicity (49); cells were then placed back in ARV-containing media in the presence of IL-2. After 48 h, cells were stained for viability, CD4 surface expression, and intracellular expression of p24-Gag as indicated below.

### Calculation of provirus inducibility.

The percentage of inducible proviruses was determined by converting the percentage of p24-positive CD4-negative cells under the CD3/CD28- or LRA-stimulated condition to reactivated cells per million cultured T_CM_ cells and divided by the copies of either total *gag* per million cultured T_CM_ cells, total intact copies per million cultured T_CM_ cells, or HMW total or intact copies per million cultured T_CM_ cells.

### Flow cytometry analysis.

For measuring latency reversal and HIV-1 infection by flow cytometry, cells were first stained with fixable viability dye (eFluor 450; eBioscience), followed by CD4 surface staining (S3.5, allophycocyanin [APC] conjugate; Life Technologies). Cells were then fixed and permeabilized as previously described (19). We define infected or reactivated cells by their CD4 downregulation and p24-Gag expression. To determine the CD4-negative p24-Gag-positive gate, uninfected donor cells were used in all experiments in parallel. Flow cytometry was performed on a Becton, Dickinson, LSR Fortessa flow cytometer using FACSDiva acquisition software (Becton, Dickinson). FlowJo software (Tree Star) was utilized to analyze data.

### Statistics.

Wilcoxon matched-pairs signed rank test or Kruskal-Wallis test with Dunn’s *post hoc* multiple-comparison test was used to calculate *P* values where indicated. Spearman correlation was calculated for correlations. Statistics were calculated using Prism 8 for Mac OS X software (GraphPad). Where indicated, calculated *P* values were adjusted for multiple comparisons using the step-down method of Holm in SAS version 9.4.

### Blood donor information.

Deidentified buffy coats were purchased from Gulf Coast Regional Blood Center. Blood donors were at least 17 years old at the time of blood donation and were HIV-1 negative. Age and gender information was available.
